# 626. Antibiotic Stewardship Practices in hospitalized patients with Dengue in South India

**DOI:** 10.1093/ofid/ofad500.692

**Published:** 2023-11-27

**Authors:** Aishwarya Balasubramaniam, Sureshkumar Dorairajan, Yogavigneshwaran Chellapandian

**Affiliations:** Vels Institute of Science, Technology and Advanced studies, Chennai, Tamil Nadu, India; Apollo Hospitals, Chennai, Tamil Nadu, India; Vels Institute of Science, Technology and Advanced studies, Chennai, Tamil Nadu, India

## Abstract

**Background:**

Antibiotics are commonly prescribed for upper respiratory viral infections, including COVID–19 infections worldwide. However, in the developing world, tropical viral infections like dengue, chikungunya, and zika are common causes of hospitalization throughout the year. But antibiotic prescribing behaviors for these infections were not studied well. Here, we are reporting the antibiotic prescribing practices for hospitalized patients with dengue in South India.

**Figure:**
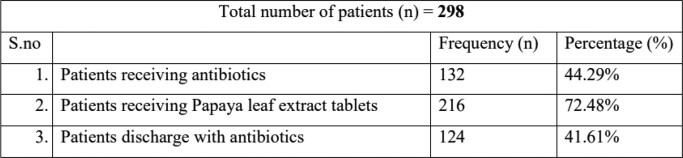

**Methods:**

We conducted a retrospective analysis during the period of October 2022 to April 2023 at multiple tertiary care hospitals, involving serologically confirmed dengue (NS-1 antigen positive) fever patients in South India. Demographic data, laboratory parameters, antibiotic prescribing practices, supportive care treatment like papaya leaf extract tablets, and antibiotics on discharge were recorded using an audit tool. Descriptive statistics were used to analyze the data and the results were tabulated.

**Results:**

During the study period 298 patients were admitted with confirmed dengue fever, other findings are listed in Table 1

**Conclusion:**

Antibiotics were prescribed to 44% of hospitalized dengue patients and 41% of the patients were discharged with antibiotics. Doxycycline and cefixime were commonly prescribed antibiotics for the patients. Most (72%) of the dengue patients were receiving papaya leaf extract as a platelet-stimulating agent despite no evidence. In the future, antimicrobial stewardship practices in the developing world should focus on tropical infections apart from bacterial and fungal infections.

**Disclosures:**

**All Authors**: No reported disclosures

